# Conference Calendar

**DOI:** 10.1002/cfg.410

**Published:** 2004-06


**Comparative and Functional Genomics**
*Comp Funct Genom* 2004; **5**: 370–372.
Published online in Wiley InterScience (www.interscience.wiley.com). **DOI:** 10.1002/cfg.410

### Conference Calendar

October–December 2004

### Animal models

Chicken Genome Sequence: Impact and Applications Symposium, 13–14 November

**http://www.avigenics.com/symposium1.htm**

### Bacteria

1st International Conference on Environmental, Industrial and Applied Microbiology (BioMicroWorld2004), 13–16 December

**http://www.formatex.org/biomicroworld2004/index.htm**

### Bioinformatics

IBC — BioDigital 2004, 13–15 October

**http://www.biodigital.de/**

NAS — Arthur M. Sackler Colloquium: Frontiers in Bioinformatics: Unsolved Problems and Challenges, 15–17 October

**http://www4.nationalacademies.org/nas/nashome.nsf/urllinks/NAS-58MTTC?OpenDocument**

TIGR/JAX — 7th Annual Conference on Computational Genomics, 21–24 October

**http://www.tigr.org/conf/cg/**

EBI/JAX/UPENN — Standards and Ontologies for Functional Genomics, 23–26 October

**http://www.jax.org/courses/events/coursedetails.do?id=44**

CODATA — 19th International Conference: The Information Society: New Horizons for Science, 7–10 November

**http://www.codata.org/04conf/index.html**

CSHL — Genomics Workshop: Identification of Functional Elements in Mammalian Genomes, 11–13 November

**http://meetings.cshl.org/2004/2004meetings.htm**

WSEAS — 5th International Conference on Mathematics and Computers in Biology and Chemistry, 15–17 November

**http://www.worldses.org/conferences/2004/iran/mcbc/index.html**

### Functional genomics

ASM — Functional Genomics and Bioinformatics Approaches to Infectious Disease Research, 6–9 October

**http://www.asm.org/Meetings/index.asp?bid=22657**

EMBO/EMBL — Functional Genomics: Exploring the Edges of Omics, 16–19 October

**http://www.embl.de/conferences/Omics04/**

CHI — Functional Genomics, 9–10 November

**http://www.healthtech.com/2004/fgn/index.asp**

IBC — EuroTIDES 2004, 22–24 November

**http://www.ibc-lifesci.com/eurotides/**

### General

ASHG — 54th Annual Meeting, 26–30 October

**http://genetics.faseb.org/genetics/ashg/menu-annmeet.shtml**

ASCB — 44th Annual Meeting, 4–8 December

**http://www.ascb.org/meetings/am2004/main04mtg.htm**

Copyright © 2004 John Wiley & Sons, Ltd.

### Metabolomics

CHI — Metabolic Profiling, 13–15 December

**http://www.healthtech.com/conference-fall04.asp**

CHI — Drug Metabolism, 13–15 December

**http://www.healthtech.com/conference-fall04.asp**

### Pharmacogenomics

3rd Annual Meeting of the International Society of Pharmacogenomics (ISP), 30 September–4 October

**http://biol.prospective-conf.u-nancy.fr/Welcome.htm**

1st International Conference on Basic and Clinical Immunogenomics, 3–7 October

**http://www.diamond-congress.hu/bci2004/**

IBC — Drug Discovery Technology: Asia-Pacific, 18–20 October

**http://www.drugdisc.com/asiapacific**

CHI — Target Validation, 11–12 November

**http://www.healthtech.com/2004/tvd/index.asp**

CSHL/WT — Pharmacogenomics, 18–21 November

**http://meetings.cshl.org/2004/2004pharm.htm**

### Plants

CSHL — Plant Genomes: From Sequence to Phenome, 9–12 December

**http://meetings.cshl.org/2004/2004plants.htm**

### Proteomics

AACR — Advances in Proteomics in Cancer Research, 6–10 October

**http://www.aacr.org/2004Proteomics1.asp**

HUPO — 3rd Annual World Congress: Proteomics: Decoding the Genome, 25–27 October

**http://www.hupo2004.cn**

### Structural genomics

EMBO — Conference on Structures in Biology, 10–13 November

**http://www.embl-heidelberg.de/conferences/StructBiol04/**

3rd International Conference on Structural Genomics, 17–21 November

**http://www-nmr.cabm.rutgers.edu/icsg2004/**

### Systems biology

5th International Conference on Systems Biology, 9–13 October

**http://www.icsb2004.org/**

### Transcriptomics

CSHL — Dynamic Organization of Nuclear Function, 29 September–3 October

**http://meetings.cshl.org/2004/2004nucleus.htm**

### Biotech industry

BioTechnica Asia, 12–14 October

**http://www.biotechnica-asia.com**

Biotechnology 2004, 17–22 October

**http://www.conicyt.cl/IBS2004/**

### Key

AACR — American Association for Cancer Research: **http://www.aacr.org**
ASCB — American Society for Cell Biology: **http://www.ascb.org/**

Copyright © 2004 John Wiley & Sons, Ltd.

ASHG — American Society for Human Genetics: **http://www.ashg.org**
ASM — American Society for Microbiology: **http://www.asm.org**
CHI — Cambridge Healthtech Institute: **http://www.healthtech.com**
CODATA — The International Council for Science Committee on Data for Science and Technology: **http://www.codata.org/**
CSHL — Cold Spring Harbor Laboratories: **http://meetings.cshl.org/index.htm**
EBI — European Bioinformatics Institute: **http://www.ebi.ac.uk**
EMBL — European Molecular Biology Laboratory: **http://www.embl-heidelberg.de/**
EMBO — European Molecular Biology Organization: **http://www.embo.org/**
HUPO — Human Proteome Organization: **http://www.hupo.org/**
IBC — IBC Conferences: **http://www.ibc-lifesci.com**
JAX — Jackson Laboratories: **http://www.jax.org/**
NAS — National Academy of Sciences: **http://www4.nationalacademies.org/nas/nashome.nsf**
TIGR — The Institute for Genomic Research: **http://www.tigr.org**
UPENN — University of Pennsylvania: **http://www.upenn.edu**
WSEAS — World Scientific and Engineering Academy and Society: **http://www.worldses.org**
WT — Wellcome Trust: **http://www.wellcome.ac.uk/**

The Conference Calendar is a listing of forthcoming conferences of interest to our readership. We provide the title, dates and web address of each conference, to enable readers to find out more information. Inclusion does not imply endorsement by the journal. If you know of a relevant conference, please contact the Managing Editor.

Copyright © 2004 John Wiley & Sons, Ltd.

